# An evaluation of barriers to the initiation of clozapine in patients with treatment-resistant schizophrenia

**DOI:** 10.1192/bjo.2021.135

**Published:** 2021-06-18

**Authors:** Declan Hyland, Seth Jamieson

**Affiliations:** 1 Consultant Psychiatrist, Clock View Hospital, Liverpool, Mersey Care NHS Foundation Trust; 2 3rd year medical undergraduate, University of Liverpool

## Abstract

**Aims:**

This evaluation aimed to identify patient, practitioner and infrastructural barriers to initiation of clozapine treatment in patients with treatment-resistant schizophrenia (TRS). In response to recent research supporting use of clozapine as the most effective treatment for patients with TRS, concerted efforts have been made to establish why clozapine is underutilised in the NHS. Following a study conducted by South London and Maudsley NHS Foundation Trust, which identified barriers and made recommendations, this evaluation aimed to identify barriers to initiation of clozapine in patients under the care of Mersey Care NHS Foundation Trust.

This evaluation also aimed to make further recommendations to increase use of clozapine in Mersey Care's TRS patients and assess whether there have been any differences to concerns about clozapine initiation compared to previous evaluations.

**Method:**

An online questionnaire containing a series of Likert scales was e-mailed to all Consultant Psychiatrists in Mersey Care NHS Foundation Trust. The questionnaire asked Consultants to rate how often they felt a range of barriers interfered with successful initiation of Clozapine treatment. The barriers chosen were based on the 2019 systematic review “Barriers to using clozapine in treatment-resistant schizophrenia.”

**Result:**

Nineteen consultant psychiatrists completed the online questionnaire. All 19 indicated they either “agreed” (16%) or “strongly agreed” (84%) that they were confident in diagnosing TRS. This was a significant increase compared to the South London and Maudsley evaluation, with only 81% of participants in that study being “fairly familar” or “very familiar” with clozapine guidelines.

Furthermore, concerns about inadequate blood testing facilities appear to have been addressed, with no participants in this evaluation staing there were insufficient blood testing facilities. However, 53% of Consultants who completed this evaluation stated they “often” (37%) or “very often” (16%) have patients who refuse clozapine because of the requirement for regular blood testing. Refusal to agree to required blood testing was the commonest reason identified for failure to initiate clozapine in TRS patients. This was consistent with the results from the South London and Maudsley study.

**Conclusion:**

Those Mersey Care consultants surveyed identified that providing patients with further information about clozapine would be the most valuable intervention to increase likelihood of uptake of clozapine in the treatment of TRS. Significant progress has been made in improving the likelihood that clozapine can be successfully initiated, especially in the removal of practitioner barriers. This evaluation suggests interventions should now be aimed at reducing patient barriers to initiation of treatment.

